# Camu-Camu Reduces Obesity and Improves Diabetic Profiles of Obese and Diabetic Mice: A Dose-Ranging Study

**DOI:** 10.3390/metabo12040301

**Published:** 2022-03-29

**Authors:** Anne Abot, Amandine Brochot, Nicolas Pomié, Eve Wemelle, Céline Druart, Marion Régnier, Nathalie M. Delzenne, Willem M. de Vos, Claude Knauf, Patrice D. Cani

**Affiliations:** 1 Enterosys SAS, 31670 Labège, France; anne.abot@enterosys.com (A.A.); nicolas.pomie@enterosys.com (N.P.); 2 A-Mansia Biotech SA, The Akkermansia Company, 1435 Mont-Saint-Guibert, Belgium; amandine.brochot@a-mansia.com (A.B.); celine.druart@a-mansia.com (C.D.); 3 Institut National de la Santé et de la Recherche Médicale (INSERM), U1220, Institut de Recherche en Santé Digestive et Nutrition (IRSD), Université Paul Sabatier (UPS), 31000 Toulouse, France; eve.wemelle@inserm.fr; 4 NeuroMicrobiota Lab, International Research Program (IRP) INSERM, 31000 Toulouse, France; 5 WELBIO—Walloon Excellence in Life Sciences and BIOtechnology, Metabolism and Nutrition Research Group, Louvain Drug Research Institute, Université Catholique de Louvain (UCLouvain), 1200 Brussels, Belgium; marion.regnier@uclouvain.be (M.R.); nathalie.delzenne@uclouvain.be (N.M.D.); 6 Laboratory of Microbiology, Wageningen University, 6708 WE Wageningen, The Netherlands; willem.devos@wur.nl; 7 Human Microbiome Research Program, Faculty of Medicine, University of Helsinki, 00014 Helsinki, Finland

**Keywords:** Camu-Camu extract, nutraceuticals, obesity, diabetes, antioxidant

## Abstract

Overweight, obesity, and their comorbidities are currently considered a major public health concern. Today considerable efforts are still needed to develop efficient strategies able to attenuate the burden of these diseases. Nutritional interventions, some with plant extracts, present promising health benefits. In this study, we evaluated the action of Camu-Camu (*Myrciaria dubia*), an Amazonian fruit rich in polyphenols and vitamin C, on the prevention of obesity and associated disorders in mice and the abundance of *Akkermansia muciniphila* in both cecum and feces. Methods: We investigated the dose-response effects of Camu-Camu extract (CCE) in the context of high-fat-diet (HFD)-induced obesity. After 5 weeks of supplementation, we demonstrated that the two doses of CCE differently improved glucose and lipid homeostasis. The lowest CCE dose (62.5 mg/kg) preferentially decreased non-HDL cholesterol and free fatty acids (FFA) and increased the abundance of *A. muciniphila* without affecting liver metabolism, while only the highest dose of CCE (200 mg/kg) prevented excessive body weight gain, fat mass gain, and hepatic steatosis. Both doses decreased fasting hyperglycemia induced by HFD. In conclusion, the use of plant extracts, and particularly CCE, may represent an additional option in the support of weight management strategies and glucose homeostasis alteration by mechanisms likely independent from the modulation of *A. muciniphila* abundance.

## 1. Introduction

Overweight, obesity, and their comorbidities (i.e., type 2 diabetes [T2D], hepatic steatosis, hyperlipidemia, and cardiovascular diseases) are currently considered a global pandemic [1]. With more than one-third of the adult population affected worldwide, obesity-associated metabolic disorders have become a major public health concern. The predictions indicate that one in five adults worldwide is expected to be affected by obesity by 2025 [2]. The same wave is predicted for T2D, with 417.3 million people suffering from diagnosed or undiagnosed T2D by 2030 [3]. These diseases are spreading in all parts of the world and are especially on the rise in developing and newly developed nations [3]. The exact relationship between overweight and fasting hyperglycemia remains somewhat of a “chicken or egg” controversy, but the link between obesity and T2D was recognized as early as the 1920s [4]. Insulin resistance is a pre-requisite for the appearance of metabolic syndrome (i.e., overweight, dyslipidemia, and hypertension) [5,6]. Other evidence suggests that visceral adiposity triggers systemic insulin resistance [7]. Indeed, overweight and T2D frequently occur together, and the vast majority of T2D patients are or have been overweight.

Generally, obesity is a consequence of an energy imbalance between intake and expenditure, lifestyle, and Western diet habits (high fat and low fiber content). However, the hallmarks of obesity and T2D are more complex. Both have been described as a possible result of chronic low-grade inflammation and the disruption of the integrity of the intestinal barrier, notably characterized by a reduction of tight junction proteins and protective mucus production. This increased intestinal permeability may allow the translocation of the pro-inflammatory bacterial compounds, such as lipopolysaccharides (LPS), responsible for metabolic endotoxemia and which gradually lead to chronic low-grade inflammation and insulin resistance [8]. Growing evidence suggests that the gut microbiota is also an important factor in the pathogenesis of obesity and the alteration of glucose homeostasis [9,10,11,12]. For example, a mucus-utilizing bacterium, *Akkermansia muciniphila* (*A. muciniphila*), which is naturally present in the gut, possesses confirmed health-promoting effects in both rodents and humans by different mechanisms [13,14,15]. Its abundance is decreased during obesity [16] and diabetes [17,18] but increased following prebiotic administration [14,19]. In rodents and humans, the administration of the bacteria (alive or pasteurized) is significantly associated with the improvement of cardiometabolic parameters [13,15,20].

Modifications of hygienic-dietetic habits are generally advised to support weight loss or glycemia control, but these lifestyle modifications are generally hard to maintain in the long term [21]. To help people obtain a healthier lifestyle, scientifically and clinically proven functional ingredients are needed with respect to consumers’ demands: natural, safe, effective, and sustainably produced. Amongst the possible options, different plant extracts could be of interest. Various studies demonstrated that plant extracts containing phytochemicals, including polyphenols, exert beneficial biological effects, including anti-diabetic and anti-obesity activities [22,23,24,25,26,27] via different mechanisms: anti-oxidant, anti-inflammatory, immunomodulatory, and through the modulation of the gut microbiota composition and/or microbially derived phenolic metabolites [28,29,30].

A potential candidate is Camu-Camu (*Myrciaria dubia*, CC). CC is an Amazonian fruit with potential health benefits since it contains an impressive quantity of vitamin C (nearly 6 g/100 g of fresh pulp), making it one of the richest sources of this vitamin in the world [31]. CC also contains polyphenols, which are well documented for their anti-oxidant and anti-inflammatory activities that are associated with improvements in body weight gain, glucose metabolism, and hepatic steatosis in rodents [28,31,32,33,34]. A recent study demonstrated an anti-obesity effect of CCE with a beneficial impact on lower body weight, fat mass gain, triglyceride accumulation in the liver, and higher energy expenditure (i.e., measured in metabolic cages and browning/beiging processes) upon high-fat and high-sucrose fed treatments [28]. In this study it was suggested that the increase in *A. muciniphila* could contribute to the phenotype observed. However, there is currently no investigation of either the impact of CCE in a diet-induced obesity model restricted to fat to induce massive obesity, or of a dose-ranging effect. The objective of this study was to investigate the impact of the administration of two different doses of CCE on obesity, on *A. muciniphila* levels in the gut, and on metabolic disorders induced by a high-fat diet.

## 2. Results

### 2.1. High Dose of Camu-Camu Prevents Obesity in Diet-Induced Obese Mice

**Figure 1:**
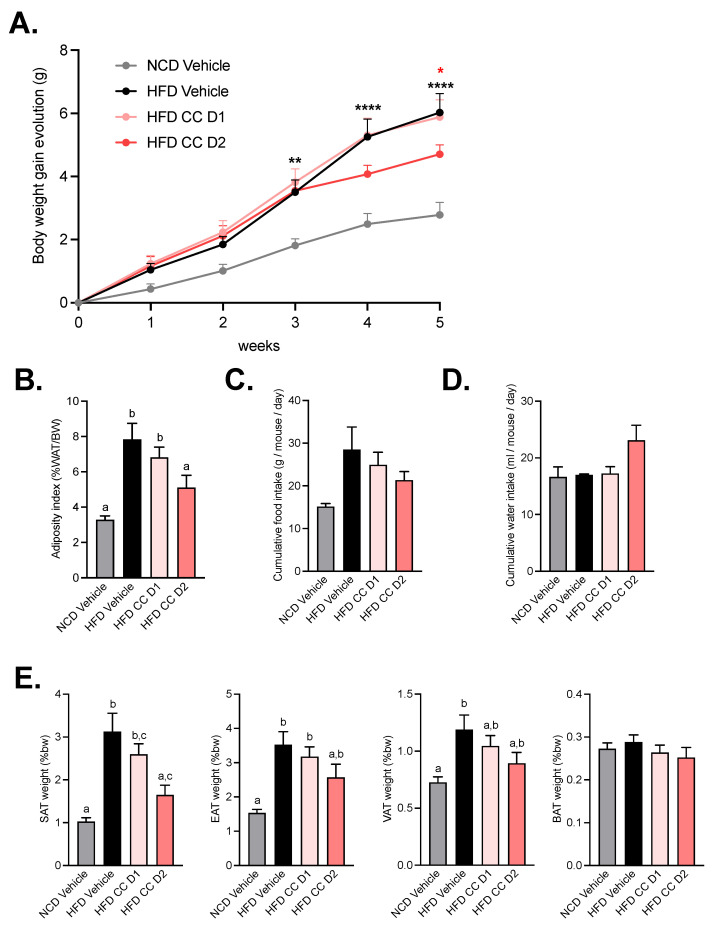
Camu-Camu prevents obesity in diet-induced obese mice only at high dose of treatment. Effects of oral administration of vehicle or CCE on (**A**) body weight gain evolution, * *p* < 0.05, ** *p* < 0.01, **** *p* < 0.0001 vs. HFD vehicle based on 2-way ANOVA followed by Bonferroni’s post hoc test, (**B**) adiposity index (% of total white adipose tissue mass divided by the body weight), (**C**) cumulative food intake, (**D**) cumulative water intake, (**E**) relative fat-mass distribution (% of body weight) of subcutaneous adipose tissue (SAT), epididymal adipose tissue (EAT), visceral adipose tissue (VAT), and brown adipose tissue (BAT). Per group, *n* = 9–10. In figures (**B**,**E**) data with different superscript letters are significantly different (*p* < 0.05) according to post hoc ANOVA one-way statistical analysis (Bonferroni’s post hoc test). In panel (**A**), only statistics vs. HFD vehicle were presented in the graph. The significant comparisons were: ** *p* < 0.01 at week 3, and **** *p* < 0.0001 at week 4 and 5 for HFD vs. NCD vehicle. and * *p* < 0.05 at week 5 for HFD CCE D2 vs. HFD vehicle.

As expected, mice fed chronically with a high-fat diet (HFD) developed obesity from the third week until the end of the experiment compared with mice fed a normal, control diet (NCD) (Figure 1A,B). CCE, only at the highest dose (CCE D2), decreased body weight gain. This observation was associated with a reduced adiposity index (Figure 1B). These improvements were not associated with changes in food or water intake (Figure 1C,D). However, the lower body weight was linked to lower subcutaneous adipose tissue (Figure 1E), whereas the other fat pads (visceral and epididymal) were decreased by about 25% but did not reach significance (*p* = 0.2175 for visceral adipose tissue and *p* = 0.2025 for epididymal adipose tissue).

### 2.2. Camu-Camu Improves Glucose Tolerance in Diet-Induced Obese Mice in a Dose-Dependent Manner

**Figure 2:**
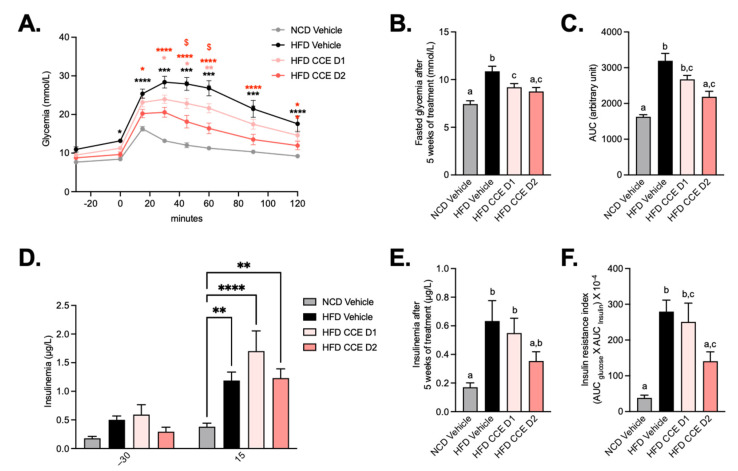
Camu-Camu improves glucose tolerance in diet-induced obese mice in a dose-dependent manner. Effects of oral administration of vehicle or CCE in 6-hour-fasted mice on (**A**) oral glucose tolerance test (OGTT), glycemia before and after an oral load of glucose (2 g/kg of body weight) after 4 weeks of treatment * *p* < 0.05, ** *p* < 0.01, **** *p* < 0.0001 vs. HFD vehicle and $ *p* < 0.05 HFD CCE D1 vs. HFD CCE D2 based on 2-way ANOVA followed by Bonferroni’s post hoc test, (**B**) fasted glycemia at the necropsy after 5 weeks of treatment, (**C**) glucose area-under-the-curve (AUC) measured during the OGTT after 4 weeks of treatment, (**D**) insulinemia 30 min before and 15 min after an oral load of glucose after 4 weeks of treatment, ** *p* < 0.01, **** *p* < 0.0001 vs. HFD vehicle based on 2-way ANOVA followed by Bonferroni’s post hoc test, (**E**) insulinemia at the necropsy after 5 weeks of treatment, (**F**) insulin resistance index determined by multiplying the AUC of blood glucose by the AUC of insulin between 30 min before and 15 min after glucose loading after 4 weeks of treatment, *n* = 9–10 per group. In figures (**B**,**C**,**E**,**F**), data with different superscript letters are significantly different (*p* < 0.05) according to post hoc ANOVA one-way statistical analysis (Bonferroni’s post hoc test). In panel (**A**), only statistics vs. HFD vehicle were presented in the graph. The other significant comparisons were: *** *p* < 0.001 after 15 min, **** *p* < 0.0001 after 30 min, **** *p* < 0.0001 after 45 min, **** *p* < 0.0001 after 60 min, **** *p* < 0.0001 after 90 min, and ** *p* < 0.01 after 120 min for HFD CCE D1 vs. NCD vehicle and **** *p* < 0.0001 after 30 min, ** *p* < 0.01 after 45 min, and * *p* < 0.05 after 60 min for HFD CCE D2 vs. NCD vehicle.

As expected, glucose tolerance was impaired in HFD-fed mice and fasting glycemia remained higher at the end of the experience, thereby confirming the diabetic status of these mice (Figure 2A,B). This effect was attenuated after supplementation with D1 and D2 of CCE but more strictly by the highest dose, suggesting a dose-effect of CCE on glucose tolerance (Figure 2A–C). The highest dose of CCE (CCE D2) significantly decreased the glycemia peak starting from T + 15 min, whereas CCE D1 decreased blood glucose between 30 and 60 min after the oral glucose challenge as compared with the HFD (Figure 2A). Mice fed an HFD logically exhibited insulin resistance, but insulin levels during OGTT and at the end of the experiment were unchanged after CCE supplementation compared with HFD (Figure 2D,E). However, the insulin resistance index was significantly lower after supplementation with the highest dose of CCE compared with both vehicle groups (Figure 2F).

### 2.3. Camu-Camu Has a Slight Impact on Hepatic Metabolism in HFD Mice

**Figure 3:**
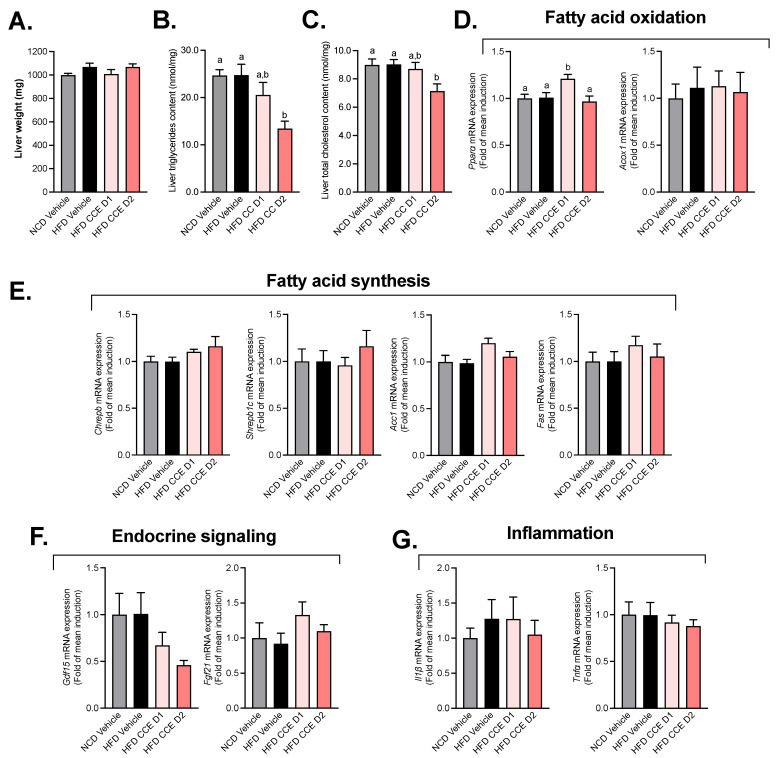
Effect of Camu-Camu on hepatic metabolism in DIO mice. Effects of oral administration of vehicle or CCE in 6-hour-fasted mice after 5 weeks of treatment on (**A**) liver weight, (**B**) liver triglycerides, and (**C**) cholesterol content and the gene expression of hepatic biomarkers involved in (**D**) fatty acid oxidation (no significant difference was observed), (**E**) fatty acid synthesis, (**F**) endocrine signaling (no significant difference was observed), (**G**) inflammation (no significant difference was observed); *n* = 9–10 per group. In figure (**B**–**D**), data with different superscript letters are significantly different (*p* < 0.05) according to post hoc ANOVA one-way statistical analysis (Bonferroni’s post hoc test).

To further explore whether the improved glucose metabolism could be linked to liver metabolism, we measured the total liver weight and liver triglycerides and cholesterol content as a marker of steatosis. First, we did not observe any difference in the liver weight (Figure 3A) upon the high-fat diet (HFD) treatment nor after the CCE treatments. However, we found a significant decrease in both triglycerides and cholesterol content in mice treated with the higher dose of CCE (Figure 3B,C). We further quantitatively measured the mRNA-expression levels of genes involved in fatty acid metabolism (fatty acid synthesis or oxidation), endocrine signaling, and the inflammation state in the liver of fasted mice after 5 weeks of treatment (Figure 3). Among the genes involved in FA oxidation, the lowest dose of CCE (CCE D1) was associated with a significant increase in *Pparα* mRNA expression (Figure 3D). We did not observe any impact of CCE on the studied biomarkers involved in fatty acid synthesis (Figure 3E). Other biomarkers of hepatic function (endocrine signaling and inflammation) were not modulated in response to CCE treatment (Figure 3F,G).

### 2.4. Camu-Camu Improves Plasma Lipid Profile in Diet-Induced Obese Mice

**Figure 4:**
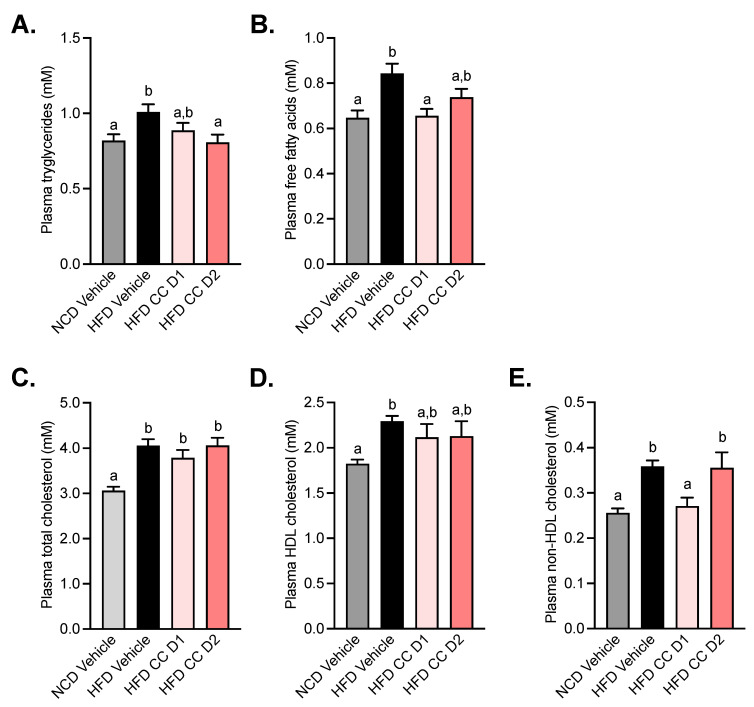
Camu-Camu improves plasma lipid profile in diet-induced obese mice. Effects of oral administration of vehicle or CCE in 6-hour-fasted mice after 5 weeks of treatment on (**A**) plasma triglycerides, (**B**) plasma free fatty acids, (**C**) plasma total cholesterol, (**D**) plasma HDL cholesterol, (**E**) plasma non-HDL cholesterol; *n* = 9–10 per group. In figures (**A**–**E**) data with different superscript letters are significantly different (*p* < 0.05) according to post hoc ANOVA one-way statistical analysis (Bonferroni’s post hoc test).

After 5 weeks of treatment, plasma concentrations of lipids were assessed (Figure 4). We observed an increase in triglycerides, free fatty acids, and cholesterol (total, HDL, and non-HDL) levels in the plasma of HFD mice. The oral administration of the highest dose of CCE (CCE D2) induced a significant decrease in triglyceride levels (Figure 4A). On the other hand, the lowest dose of CCE (CCE D1) had a beneficial impact on free fatty acid and non-HDL cholesterol levels (Figure 4B,E).

### 2.5. Camu-Camu Supplementation Increases Adipose Tissue Triglyceride Hydrolases without Affecting Markers of Browning in the Subcutaneous Adipose Tissue

**Figure 5:**
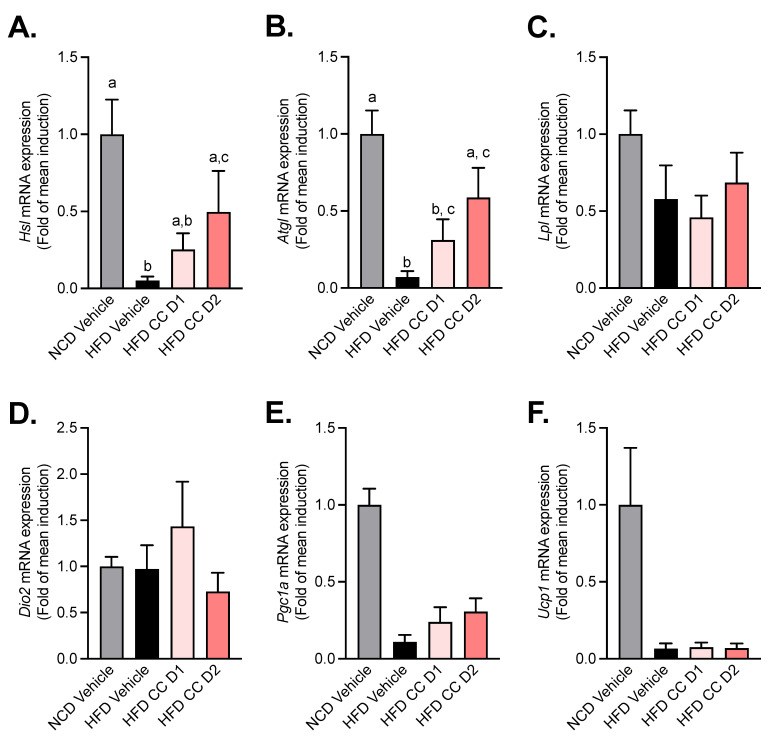
Camu-Camu reduces HFD-induced decrease in markers of triglyceride hydrolases without affecting markers of browning in the subcutaneous adipose tissue. Effects of oral administration of vehicle or CCE in 6-hour-fasted mice after 5 weeks of treatment on the mRNA expression of (**A**) hormone-sensitive lipase (*Hsl*) and (**B**) adipose triglyceride lipase (*Atgl*). (**C**) Lipoprotein lipase (*lpl*) and (**D**–**F**) the gene expression of markers of browning (*Dio2*, *Pgc1a*, *Ucp1*) (no significant difference was observed); *n* = 5–9 per group. In figure (**A**,**B**), data with different superscript letters are significantly different (*p* < 0.05) according to post hoc ANOVA one-way statistical analysis (Bonferroni’s posthoc test).

Given that CCE reduces subcutaneous fat mass, plasma triglycerides, and liver steatosis we measured several markers related to triglyceride hydrolysis, oxidation, and browning. Adipose triglyceride lipase (*Atgl*) and hormone-sensitive lipase (*Hsl*) are key enzymes involved in the intracellular degradation of triacylglycerols. We found that CCE administration dose-dependently increases the mRNA expression of both *Atgl* and *Hsl*, with a significant and stronger effect with the highest dose of CCE (CCE D2) (Figure 5A,B). Strikingly, the other markers, involved mainly in lipolysis (*Lpl*) and browning (i.e., *dio2*, *UCP-1*, *PGC1-a*), were mostly affected by the HFD but not by the CCE treatment (Figure 5C–F).

### 2.6. Camu-Camu Supplementation Attenuates the Effect of HFD on the Abundance of Akkermansia Muciniphila

**Figure 6:**
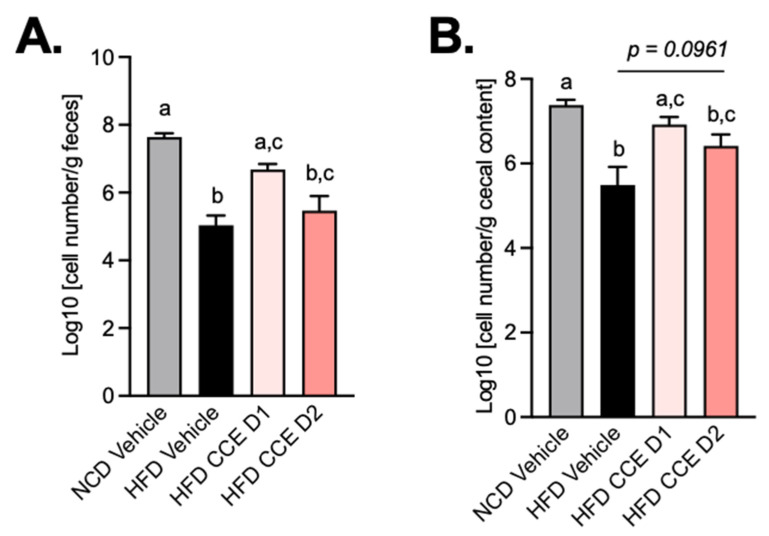
Camu-Camu prevents *A. muciniphila* decrease induced by HFD. Effects of oral administration of vehicle or CCE on quantification of *A. muciniphila* (**A**) in feces, (**B**) in cecal content.; *n* = 9–10 per group. In figures (**A**,**B**), data with different superscript letters are significantly different (*p* < 0.05) according to post hoc ANOVA one-way statistical analysis (Bonferroni’s post hoc test).

Given that it has been previously proposed that CCE administration specifically increased the levels of *A. muciniphila* in the gut microbiota of mice and that the metabolic effects could be linked to this bacterium [28], we measured the abundance of this beneficial bacteria by qPCR at the end of the treatment period in both feces and cecal content. We found that HFD treatment significantly decreased *A. muciniphila* abundance in both the feces and the cecal content, whereas the lowest dose of CCE (CCE D1) significantly increased *A. muciniphila* compared with HFD-treated mice. Strikingly, this increase of *A. muciniphila* abundance did not reach significance in response to the highest dose of CCE (CCE D2) (*p* = 0.0961) (Figure 6A,B). Therefore, these data suggest that the changes in the levels of *A. muciniphila* observed upon CCE treatment could not be explained by the improved metabolic phenotype.

## 3. Discussion

The main goal of the study was to evaluate the dose-ranging effect of CCE in a preclinical model of obesity and its impact on *A. muciniphila* levels. We have demonstrated that two different doses of CCE (62.5 mg/kg/day and 200 mg/kg/day) differently improved glucose and lipid homeostasis and that these effects were not directly explained by a dose–response effect on *A. muciniphila*. Interestingly, we found that the lowest dose preferentially decreased non-HDL cholesterol and FFA with no effect on hepatic liver weight nor on liver fatty-acid metabolism while increasing the abundance of *A. muciniphila*. However, we discovered that only the highest dose of CCE prevented body weight gain, fat mass gain, liver triglyceride accumulation, liver cholesterol accumulation, hyperinsulinemia, and insulin resistance induced by the HFD. Also, we noticed that both doses of CCE were efficient in decreasing hyperglycemia and improving glucose tolerance. These results corroborate previous findings from Anhé et al. and extend knowledge about the dose-ranging effect of CCE in response to an HFD on metabolic syndrome and the levels of *A. muciniphila* in the gut [28]. In this last study, the authors found that a daily administration of CCE at 200 mg/kg/day prevented the body weight gain induced by a high-fat–high-sucrose (HF-HS) diet and reduced the adiposity index by impacting, specifically, the subcutaneous adipose tissue. As in our experiment, they found that the anti-obesity effect of CCE was distinct from changes in energy intake as no impact on food intake was demonstrated. However, they could explain the effects of CCE on body weight and fat mass by a mechanism linked to an increased energy expenditure and adaptive thermogenesis [28]. Indeed, Anhe et al. demonstrated that CCE ingestion was associated with higher energy expenditure, which could be partially explained by a higher expression of the key thermogenic marker UCP-1 (uncoupling protein 1) in brown adipose tissue. Moreover, they found that CCE-treated mice exhibited a specific bile acid profile which could also contribute to activating the membrane bile acid receptor TGR-5 [28]. Whether this mechanism explains the overall prevention in weight gain remains to be proven. Indeed, in our study, none of the doses used could be linked with any of the markers of adipose tissue browning (i.e., *dio2*, *Ucp1*, and *Pgc1a*) that remained unaffected by the treatment.

The lowest dose of CCE had no effect on body weight gain and adiposity, and we found that supplementing mice with a low dose of CCE for 5 weeks was sufficient to decrease fasted glycemia, meaning that, at this dose, CCE extract could have a positive impact on fasted glycemia without affecting the prevention of body weight gain. Compared with the lower dose of CCE, the higher dose significantly reduced body weight gain and also increased the benefits of CCE on glycemia throughout the OGTT. Strong evidence supports the association between post-prandial hyperglycemia, increased risk of T2D, elevation of HbA1c, and metabolic syndrome [35,36]. Reduced blood glucose levels after a bolus of glucose or a carbohydrate-rich meal could be of interest in preventing the development of chronic hyperglycemia. One of the strategies to decrease post-prandial hyperglycemia is the inhibition of two enzymes, α-amylase and α-glucosidase, linked to soluble carbohydrate digestion and associated glucose metabolism. Previous in vitro studies have shown the potential of phenolic-rich plant-based food extracts for inhibition of these enzymes [30,37,38,39]. A recent experimental study confirmed that CCE powders were highly effective in inhibiting α-glucosidase [40] and no other fruits analyzed, such as strawberry (high inhibition at 25 mg/mL), tea (600 μg/mL), raspberry (200 μg/mL), or pear (500 mg/mL) possessed the same inhibitory potential as CCE (50 μg/mL). Furthermore, the effect of CCE on post-prandial glycemia was confirmed in healthy humans. In this study, volunteers were told to consume a test meal consisting of 50 g white bread (25 g of available carbohydrate) plus 300 mL of water or clarified CCE juice [41]. Post-prandial blood glucose levels were significantly lower after consumption of the test meal with CCE juice compared with water [41]. The natural enzyme inhibitors from fruits and vegetables could provide health benefits without any side effects such as flatulence, diarrhea, and abdominal distention typically caused by drugs such as acarbose [38,42,43,44], a reversible inhibitor of pancreatic α-amylase and membrane-bound intestinal α-glucoside hydrolase.

The evaluation of the parameters of dyslipidemia, demonstrated that plasma non-HDL and free fatty acid levels were significantly decreased with the lowest dose of CCE. The highest dose of CCE decreased only the plasma triglyceride level. Previous studies have already reported the effects of CCE on lipid parameters, but none described a different effect on the lipid profile depending on the dose administrated [28,45]. It has been reported in the literature that CC fruit was able to inhibit the hepatic synthesis of VLDL and to increase activity of the enzyme lipoprotein lipase (LPL), which could result in a reduction in plasma triglycerides, mainly in the post-prandial state [31]. However, we found that CCE dose-dependently increased the expression of two key enzymes involved in triglyceride hydrolysis, namely adipose triglyceride lipase (ATGL) and hormone-sensitive lipase (HSL) [46], without affecting the expression of *Lpl.* Another mechanism involved in TG regulation was *PPARα* activation [47]. In our model, CCE at the lowest dose increased the relative mRNA expression of *Pparα* in the liver and tended to decrease hypertriglyceridemia induced by an HFD. Other than this effect on *Pparα* mRNA expression, we did not observe any other changes in hepatic weight, inflammation, or lipid or glucose metabolism. However, we must acknowledge that our assessment of liver inflammation was mainly based on mRNA analysis and did not use specific histological analysis of the liver tissue. Whether Carnitine palmitoyltransferase 1 (CPT1), the rate limiting step in long-chain fatty acid oxidation could be affected remains to be shown, as this was not investigated in our study. Nevertheless, the near total lack of changes in the overall liver markers can be explained by the fact that the duration of the dietary treatment was only 5 weeks or the dose used was too low or because the extract used has no inherent activity on these parameters. As shown in the HFD-fed mice, the liver makers were not affected by the HFD itself, except for the lower accumulation of triglycerides and cholesterol that might upon the highest dose of CCE treatment that, per se, may have already contributed to improved glucose metabolism. Moreover, we may not exclude the possibility that the decreased levels of plasma triglycerides could be explained by extrahepatic clearance, such as via higher LPL activity in the muscles and/or adipose tissues instead of as an effect on the liver. However, the later argument is likely not supported by our data since we could not find any change in the *lpl* mRNA expression in the subcutaneous adipose tissue. Therefore, one may argue that the overall metabolic improvements observed upon CCE treatment were not directly linked to hepatic inflammation or specific alteration of both lipid synthesis and oxidation in the liver but only linked to the lower triglyceride accumulation, and possibly to higher adipose tissue ATGL and HSL activity. Altogether, these findings suggest that other tissues might be impacted earlier by dietary intervention and thereby contribute to the regulation of the host metabolism (e.g., adipose tissue, muscles, and brain) [13]. Altogether, we may not rule out that the lower fat mass and body weight gain could contribute to the improved glucose metabolism. However, given that we found a lower hepatic steatosis, higher lipid hydrolysis markers in the adipose tissue, and lower plasma triglycerides, one may speculate that these different effects positively contribute to overall glucose metabolism.

A common mechanism that has been proposed in the literature and that could explain the effects of CCE on several cardiometabolic risk factors is its possible prebiotic effect on *A. muciniphila* content in feces and cecal content. Several studies have found in rodents that prebiotics, such as inulin-type fructans and some specific plant extracts containing polyphenols, could protected mice from obesity and T2D [26,27] by increasing gut *A. muciniphila* abundance [13,14,19,48,49,50]. In our study, we found that CCE, at a dose as low as 62.5 mg/kg/day increased *A. muciniphila*, whereas the highest dose was not able to do so, although it strongly improved the phenotype of the mice. Therefore, this finding challenges the putative direct link between higher *A. muciniphila* abundance induced by CCE and the health effects of CCE. Although the impact of CCE on *A. muciniphila* and metabolic improvement under an obesogenic and diabetic diet had already been described with another CCE with the same dose as the highest dose tested in our study (200 mg/kg of body weight/day), these effects have been observed in mice treated with an HF–HS diet. Whether the use of a lipid-rich diet without sucrose, as we used in our study, can explain this difference remains to be tested. Moreover, the mechanism by which CCE increased the abundance of *A. muciniphila* is poorly understood. One of the explanations is the polyphenolic-profile content of CCE, and more specifically, its richness in proanthocyanin, the prebiotic effect of which on *A. muciniphila* had already been described [51]. A second explanation is the interplay between CCE, *A. muciniphila,* and bile acids, as *A*. *muciniphila* levels positively correlated with several bile acids in circulation and with the browning of adipose tissue [28].

In any case, our data strongly suggest that CCE improves the phenotype of mice already at a low-dose, that a higher dose of CCE might prevent the weight gain and fat mass gain, and that this is not linked to changes in food intake. However, more importantly, our study questions whether CCE really acts via a mechanism linked to a specific increase of *A. muciniphila*. The fact that the cardiometabolic benefits of polyphenols are not always dependent on the blooming of *A. muciniphila* in the gut microbiota suggests that the abundance of this bacterium, despite being influenced by PACs and ellagitannins, is not an essential factor mediating the positive health impact of some polyphenols [26,27]. Finally, we may not exclude that the effects of CCE might be linked to the gut microbiota and, eventually, not via *A. muciniphila.* Indeed, Anhé et al., have elegantly shown that transferring the microbiota form CCE-treated mice into germ-free mice partially replicated the phenotype [28]. Hence, supporting the fact that the modulation of the gut microbiota using CCE clearly occurs, but that this is likely more complex than targeting only one bacterium. Further experiments using metagenomic sequencing are warranted to elucidate whether a dose response on the overall gut microbiota composition in our experiments also existed and if no other recently identified bacteria could be linked to the current effects [52,53].

Finally, although both doses showed interesting effects on different parameters, our study suggests that to further expand these findings to humans, a dose–response study could be useful. Indeed, depending on the organ targeted (e.g., liver or adipose tissue) or the metabolic effects expected (i.e., glucose, lipid, or anti-inflammatory effects), the dose chosen could be different and this will have to be linked with tolerance and the potential toxic effects of using very high-doses.

## 4. Materials and Methods

### 4.1. Animals

Nine-week-old male C57BL/6J mice (Charles River Laboratory, Saint Germain sur l’Arbresle, France) were housed in specific pathogen-free conditions and in a controlled environment (room temperature of 22 ± 2 °C, 12 h daylight cycle) with free access to food and water. After one week of acclimatization, mice were randomly assigned to one of four groups and fed a normal control diet (NCD) for mature rodents (Research Diet # D10012Mi, New Brunswick, NJ, USA) or a high-fat diet (HFD) containing protein 20% Kcal, carbohydrate 20% Kcal, and fat 60% Kcal (Research Diet # D12492i, New Brunswick, NJ, USA) for 5 weeks. Treatment started concomitantly with the introduction of the HFD and consisted of daily oral gavage of two different CCE doses (D1: 62.5 mg/kg/day and D2: 200 mg/kg/day) of resuspended CCE in a volume of 150μL for 5 weeks of HFD treatment. The highest dose was chosen based on previous study in a high-fat–high-sucrose mice model [28]. The lowest dose was calculated to have a compatible dose in humans through a single daily administration via a capsule or a tablet (using the practice guide for dose conversion between animals and humans) [54]. The CCE (Naturex—Avignon, France) was extracted from the fruit of *Myrciaria dubia* (Kunth) Mc Vaugh, containing 20% ascorbic acid (Table S1). The oral gavage was performed each day at the end of the light phase (end of afternoon). The vehicle was composed of PBS containing 2.5% glycerol. Prior to the beginning of the protocol, glycemia and insulinemia were assessed through blood samples collected from the tip of the tail vein after 6-hour fasting. Body weight and food and water intake were assessed weekly.

### 4.2. Tissues Sampling

After 5 weeks of treatment and a 6-hour fasting period, the animals were weighed, glycemia was measured and blood samples collected from the tip of the tail vein for further analysis. After blood sampling, the animals were anesthetized. After exsanguination, mice were killed by cervical dislocation. Tissue samples (liver, brown adipose tissue, epididymal adipose tissue, subcutaneous adipose tissue, visceral adipose tissue, and cecal content) were precisely dissected and weighed. Liver, subcutaneous fat, and cecal content were immersed in liquid nitrogen and stored at –80 °C for further analysis. All mouse experiments were approved by and performed in accordance with the guidelines of the local ethics committee.

### 4.3. Oral Glucose Tolerance Test

After 4 weeks of treatment, an oral glucose tolerance test (OGTT) was performed as previously described [55,56], around 12 h after the last oral administration of vehicle or treatment. Six-hour-fasted mice were given an oral glucose load (2 g glucose/kg body weight). Glycemia was measured 30 min before (T − 30) the oral glucose load (T0) and then 15, 30, 45, 60, 90, and 120 min after oral glucose load with a glucometer (Accu-Chek Active, Roche, Switzerland) with blood samples collected from the tip of the tail vein. Blood was also collected from the tail vein at T − 30 and T + 15 min to assess plasma insulin.

### 4.4. Insulin Resistance Index

Plasma insulin concentration was determined using the HTRF method, according to the manufacturer’s instructions. The insulin resistance index was determined by multiplying the area-under-the-curve for both the blood glucose (−30 to 120 min) and plasma insulin (−30 and 15 min) obtained following the OGTT [56].

### 4.5. RNA Preparation and Real-Time qPCR Analysis

Hepatic biopsies were homogenized using a Precellys tissue homogenizer (Bertin Technol., Montigny-le-Bretonneux, France), and total RNA was extracted from tissues using TRIReagent (Sigma-Aldrich, Saint Louis, MO, USA) and a GenElute Mammalian Total RNA Miniprep Kit (Sigma-Aldrich, Saint Louis, MO, USA), according to the manufacturers’ instructions. The complementary DNAs (cDNAs) were generated either using a Moloney Murine Leukemia Virus reverse transcriptase (M-MLV) with a reverse transcriptase kit (Invitrogen) and random hexamers (Invitrogen, Waltham, US). Real-time PCR was performed with a LightCycler 480 (Life Technologies, Carlsbad, CA, USA) using SYBR Green Real-Time PCR Master Mixes (Thermo Fisher Scientific, Waltham, MA, USA) and primers that were validated by testing the PCR efficiency. Gene expression was quantified using the comparative Ct (threshold cycle) method. The results were normalized to hypoxanthine-guanine phosphoribosyl transferase expression. For the subcutaneous adipose tissue, the total RNA was prepared from tissues using TriPure reagent (Roche, Basel, Switzerland). Quantification and integrity analysis of total RNA were performed by analyzing 1 μL of each sample in an Agilent 2100 Bioanalyzer (Agilent RNA 6000 Nano Kit, Agilent, Santa Clara, CA, USA). cDNA was prepared by reverse transcription of 1 μg total RNA using a reverse transcription system kit (Promega, Madison, WI). Real-time PCR was performed with the CFX Manager 3.1 software (Bio-Rad, Hercules, CA, USA) using Mesa Fast qPCR (GoTaq qPCR Master Mix, Promega, Madison, WI, USA) for detection, according to the manufacturer’s instructions. Due to unforeseen reasons, we experienced RNA degradation for some of the subcutaneous adipose tissue samples. Hence, those samples were not included in the qPCR analysis thereby leading to *n* = 5 to 9 samples per group. RPL19 was chosen as the housekeeping gene. In both tissues, the identity and purity of the amplified product were assessed by analyzing the melting curve, which was performed at the end of amplification. The sequences of the primers used for cDNA amplification in the quantitative RT-PCR experiments are listed in online Table S2.

### 4.6. Biochemical Analyses

Plasma triglycerides, total cholesterol, high- and low-density lipoproteins (HDL, non-HDL) and free fatty acids (FFA) were analyzed in blood samples collected at the necropsy using Pentra 400, following the manufacturer’s instructions.

### 4.7. Quantification of Akkermansia Muciniphila

DNA was isolated from the cecal content and stools samples collected at the end of the protocol and analyzed as previously described [14]. Briefly, DNA was isolated using a QIAamp-DNA stool minikit (Qiagen, Hilden, Germany), according to the manufacturer’s instructions. qPCR was performed with the CFX96 Bio-Rad Real-Time PCR system and CFX Manager 3.1 software (Bio-Rad, Hercules, CA, USA). The primers and probes used to detect *A. muciniphila* were based on 16S rRNA gene sequences: forward *Akkermansia muciniphila* (*A. muciniphila*), CAGCACGTGAAGGTGGGGAC, reverse *A. muciniphila*, CCTTGCGGTTGGCTTCAGAT. Each assay was performed in duplicate in the same run. The cycle threshold (CT) of each sample was compared with a standard curve made by diluting genomic DNA isolated from a pure culture of a type of strain (BCCM/LMG, Ghent, Belgium; DSMZ, Braunshweig, Germany). The data are expressed as log of bacteria per g of content.

### 4.8. Statistical Analysis

Data are expressed as means ± SEMs. Differences between the groups were assessed using one- or two-way ANOVA, followed by post hoc Bonferroni’s test. In the figures, data with different superscript letters are significantly different at *p* < 0.05, according to post hoc ANOVA statistical analyses. Data were analyzed using GraphPad Prism version 8.00 for Windows (GraphPad Software). The results were considered statistically significant when *p* < 0.05.

## 5. Conclusions

In conclusion, we demonstrated that, in a 5-week HFD mice model, a low dose of CCE is more prone to preventing lipidic disorders, while a higher dose acts on body weight, fat mass, and glucose homeostasis. The lowest dose of CCE tested increased the abundance of the bacteria *A. muciniphila* but to a level that is not sufficient to reverse all the modifications induced by an HFD.

## Data Availability

The data presented in this study are available on request from the corresponding author due to restrictions on privacy.

## Supplementary Materials

The following are available online at https://www.mdpi.com/article/10.3390/metabo12040301/s1, Table S1: Camu-camu extract specifications; Table S2: Quantitative PCR primer sequences for targeted mouse genes.
