# Supplementary material for: Camu-Camu Reduces Obesity and Improves Diabetic Profiles of Obese and Diabetic Mice: A Dose-Ranging Study
Source: Metabolites. 2022 Mar 29;12(4):301. doi: 10.3390/metabo12040301 (PMC9025096; doi:10.3390/metabo12040301)
Supplement: Supplementary file 1 [file metabolites-12-00301-s001.zip › metabolites-1611542-supplementary.pdf]

Table S1. Camu-camu extract specification.

CAMU CAMU FRUIT PE 20% ASORBIC ACID	
**Description**	Powdered extract obtained from Camu Camu fruits<br>Botanical name: *Myciaria dubia* (Kunth) Mc Vaugh<br>Plant part used: fruits<br>Extraction solvent: Water 100%<br>Native Extract Ratio: 8-10 / 1<br>100 mg of extract equivalent to an average of 720 mg of dry *Myciaria dubia* fruits
**Specifications**	Aspect: powder<br>Color: Cream to pink<br>Flavor: Characteristic<br>Solubility: Dispersible in water

Table S2. Quantitative PCR primer sequences for the targeted mouse genes.

Gene	Forward sequence	Reverse sequence
mGdf15	TGGGACCCCAATCTCACCTCT	AGCCGAGAGGACTCGAACTCA
mFgf21	AAAGCCTCTAGGTTTCTTTGCCA	CCTCAGGATCAAAGTGAGGCG
mAcox1	CAAAGAAACCCCTCCAGCC	GCTGTGTGCCGTCTGGAGT
mPpara	CCCTGTTTGTGGCTGCTATAATTT	GGGAAGAGGAAGGTGTCATCTG
mFas	GGGTTCTAGCCAGCAGAGTC	TCAGCCACTTGAGTGTCCTC
mAcc1	TGTACAAGCAGTGTGGGCTGGCT	CCACATGGCCTGGCTTGGAGGG
mChrepb	ACCTCTTCGAGTGCTTGAGCC	GTTGCACATACTGAATGTACCAGGC
mShrepb1c	GGAAGCTGTCGGGGTAGCGTC	CATGTCTTCAAATGTGCAATCCAT
mIl1b	CAACCAACAAGTGATATTCTCGATG	GATCCACACTCTCCAGCTGCA
mTnfa	GGGACAGTGACCTGGACTGT	TTCGGAAAGCCCATTTGAGT
mHprt1	GTTCTTTGCTGACCTGCTGGAT	CCCCGTTGACTGATCATTACAG
mHsl	GCTAGCCAGGCTCATCTCCT	GTTCTTGAGGTAGGGCTCGT
mAtgl	ACAGCTCCACCAACATCCAC	AGCCCTGTTTGCACATCTCT
mUcp1	GCTACACGGGGACCTACAATG	CGTCATCTGCCAGTATTTTGTT
mPgc1a	AGCCGTGACCACTGACAACGAG	GCTGCATGGTTCTGAGTGCTAAG
mDio2	AATTATGCCTCGGAGAAGACCG	GGCAGTTGCCTAGTGAAAGGT
mLpl	TCTGTACGGCACAGTGG	CCTCTCGATGACGAAGC
mRpl19	GAAGGTCAAAGGGAATGTGTTCA	CCTGTTGCTCACTTGT
